# Knockdown of astrocyte elevated gene-1 inhibits proliferation and enhancing chemo-sensitivity to cisplatin or doxorubicin in neuroblastoma cells

**DOI:** 10.1186/1756-9966-28-19

**Published:** 2009-02-15

**Authors:** Haiyan Liu, Xianrang Song, Chunxi Liu, Li Xie, Ling Wei, Ruopeng Sun

**Affiliations:** 1 Department of Pediatrics, Qilu Hospital of Shandong University, Jinan, PR China; 2 Department of Cancer Research Center, Shandong Cancer Hospital and Institute, Jinan, PR China; 3 Laboratory of Cardiovascular Remodeling and Function Research, Qilu Hospital of Shandong University, Jinan, PR China

## Abstract

**Background:**

*Astrocyte elevated gene-1 *(*AEG*-*1*) was originally characterized as a HIV-1-inducible gene in primary human fetal astrocyte. Recent studies highlight a potential role of *AEG-1 *in promoting tumor progression and metastasis. The aim of this study was to investigate if *AEG-1 *serves as a potential therapeutic target of human neuroblastoma.

**Methods:**

We employed RNA interference to reduce *AEG-1 *expression in human neuroblastoma cell lines and analyzed their phenotypic changes.

**Results:**

We found that the knockdown of *AEG-1 *expression in human neuroblastoma cells significantly inhibited cell proliferation and apoptosis. The specific downregulation induced cell arrest in the G_0_/G_1 _phase of cell cycle. In the present study, we also observed a significant enhancement of chemo-sensitivity to cisplatin and doxorubicin by knockdown of *AEG-1*.

**Conclusion:**

Our study suggests that overexpressed *AEG-1 *enhance the tumorogenic properties of neuroblastoma cells. The inhibition of *AEG-1 *expression could be a new adjuvant therapy for neuroblastoma.

## Background

Neuroblastoma is the most common solid tumor of infancy. It is thought to arise from the anomalous arrest of multi-potential embryonal cells of neural crest origin during differentiation. The disordered differentiation contributes to the pathogenesis of the disease [1]. Prognosis of neuroblastoma is in part related to tumor stage, the presence or absence of N-myc amplification, nuclear ploidy and the age of onset [2-4]. Advanced neuroblastoma in children over 1 year old has a very poor prognosis and is resistant to standard chemotherapy. Although complete or partial remissions are achieved in 74% of these children with multi-agent high-dose therapy, long-term survivors represent only 15–20% of relapsed patients [5,6]. Relapse and metastasis are the dominated negative factors for survival. New approaches to inhibit aggressiveness and increase chemo-sensitivity of neuroblastoma to anticancer agents are required.

*Astrocyte elevated gene-1 *(*AEG-1*) was originally characterized as a human immunodeficiency virus (HIV)-1-inducible gene in primary human fetal astrocyte [7,8], which is a downstream target molecule of Ha-*ras *and c-*myc *mediating their tumor promoting effects [9]. *AEG-1 *is ubiquitously expressed in numerous cell types, elevated levels have also been observed in some solid tumors including those of breast, brain and prostate [9,10]. Intriguingly, *AEG-1 *expression is elevated in diverse neoplastic conditions, it cooperates with Ha-*ras *to promote transformation, and its overexpression in Hela cells induces increased anchorage-independent growth and invasiveness and increase expression of adhesion molecules by activating the NF-κB pathway [11]. However, such studies are lacking in neuroblastoma.

Recently, we found that *AEG-1 *is also frequently overexpressed in neuroblastoma (submitted). In patients with advanced neuroblastoma, poor clinical outcome were observed related to *AEG-1 *overexpression, highlight a potential role of *AEG-1 *in promoting tumor progression and metastasis of neuroblastoma.

In the present study, we hypothesize that overexpressed *AEG-1 *enhances tumorogenic properties of neuroblastoma cells in the same manner as observed in cultured HeLa cells [11]. The inhibition of *AEG-1 *expression could be a new adjuvant therapy for neuroblastoma.

## Methods

### Cell lines and culture

Human neuroblastoma cell lines M17 and SK-N-SH (Chinese Type Culture Collection, Beijing, China) were maintained in Dulbecco's modified Eagle's medium (DMEM, Invitrogen, Carlsbad, CA, USA) supplemented with 10% heat-inactivated fetal bovine serum (FBS, Gibco, AUS) at 37°C in an atmosphere of 5% CO_2 _with humidity.

### *AEG-1*-siRNA transfection

Knockdown of *AEG-1 *expression was achieved using transfection of *AEG-1*-siRNA. *AEG-1*-siRNA1 and *AEG-1*-siRNA2 targeting nucleotides 971–991 and 1355–1375 of human *AEG-1 *mRNA sequence (GenBank Accession No. NM_178812.3) were synthesized by Genepharma (Shanghai, China) as shown in Table 1 and annealed to form siRNA duplexes according to manufacturer's instructions. Non-targeting siRNA was used to control for non-specific effects. Cells were transfected 24 hours under standard culture conditions with 100 nM siRNA duplexes using Lipofectamine™ 2000 (Invitrogen, Carlsbad, CA) following manufacturer's protocols.

**Table 1:** Targeted AEG-1 sequences and the control siRNA were chemically synthesized by Genepharma (Shanghai, China).

Name	Senquences
*AEG-1*siRNA 1	s: 5'-GACACUGGAGAUGCUAAUAUU-3' as: 5'-UAUUAGCAUCUCCAGUGUCUU-3'
*AEG-1*siRNA 2	s: 5'-GGUGAAGAUAACUCUACUGUU-3' as: 5'-CAGUAGAGUUAUCUUCACCUU-3'
Control siRNA	s: 5'-UUCUCCGAACGUGUCACGUTT-3' as: 5'-ACGUGACACGUUCGGAGAATT-3'

### Real-time RT-PCR

Fourty-eight hours after transfection, cells were harvested in TRIzol Reagent (Invitrogen) and total RNA was isolated following the manufacturer's instructions. The first strand cDNA was synthesized according to the manufacturer's instructions (TaKaRa RT kit, Dalian, China). Quantitative determination of *AEG-1 *transcript concentrations was performed by real-time RT-PCR with GAPDH as an internal control. Primers for *AEG-1 *(sense 5' GGC AAT TGG GTA GAC GAA GA 3'; antisense 5' CCT GTT TTG GAC GGG TTT TA 3') and GAPDH (sense 5' GAG TCA ACG GAT TTG GTC GT 3'; antisense 5' TTG ATT TTG GAG GGA TCT CG 3') synthesized by Sangon (Shanghai, China) and were used to measure gene expression. Amplification reaction assays were set up triplicate for each sample using the SYBR Green system (TaKaRa, Dalian, China). In order to quantify the gene expression changes, the *ΔΔCt *method was used to calculate the relative fold-changes normalized against GAPDH.

### Western blot analysis

After 48 hours of transfection, cells and supernatant of each group would be collected. Proteins were extracted after break-down of cells by SDS boiling method. Proteins were quantified by Bradford method. 50 μg of protein underwent SDS-PAGE and was transferred to PVDF membrane afterward. It was then sealed at room temperature for 2 hours. The primary antibodies, rabbit anti-human *AEG-1 *antibody (Invitrogen, Carlsbad, CA), was added at a ratio of 1:1000, and incubated overnight at 4°C. The membrane was washed with PBS. Then, the secondary antibody, mouse anti-rabbit IgG/HRP antibodies (Amersham Biosciences), was added at a ratio of 1:5000, and incubated at room temperature for 2 hours. The membrane was washed three times and reacted with chemiluminescent agent for 5 minutes. It was then ECL tabletting, exposed, and displayed. The amount of each protein sample was controlled by β-actin.

### Cell proliferation assay

M17 and SK-N-SH cells were transfected in 6-well plate. 24 hours late, the transfected cells were trypsinized and plated in 96-well plates with 1.0 × 10^3 ^cells in 100 μl of the medium and allowed to attach for 24 h, then 10 μl of MTT (5 mg/ml in PBS) was added for 4 h incubation at 37°C after 4, 24, 48, 72 h, respectively. Subsequently the formazan crystals were solubilized with 100 μl of 10% sodium dodecyl sulfate (SDS) in 0.01 M HCl for 24 h. The absorbance was measured using a Microplate Reader (Bio-rad 680, Bio-rad, USA) with a test wavelength of 570 nm and a reference wavelength of 630 nm and all experiments were performed in triplicate. The cell proliferation curve was plotted using the absorbance at each time point.

### Colonogenic assay

The number of colonies was determined as described previously [12]. Briefly, following transfection for 48 h, cells were trypsinized, counted, and seeded for the colony forming assay in 60 mm dishes at 200 cells per dish. After incubation for 14 days, colonies were stained with crystal violet and the numbers of positive cells counted. Colonies containing more than 50 cells were scored, and triplicates containing 10–150 colonies/dish were counted in each treatment.

### Flow cytometry analysis

Detection of apoptosis and cell cycle by flow cytometry was performed using the Annexin V-FITC kit (Becton Dickinson, CA, USA). The transfected cells were harvested with trypsinization, fixed with cold 70% ethanol at 4°C for 24 hours. The staining was performed according to the producer's manual. Flow cytometry (Becton Dickinson, CA, USA) was performed immediately.

### Cell viability assay

Cell viability assay was performed as described previously [12]. Cells were seeded in 96-well plates (Corning, NY, USA). After overnight culture, they were exposed to various concentrations of cisplatin or doxorubicin for 48 h in a CO_2 _incubator. MTT assay as described above was used to detect the chemo-sensitivity of cells. Absorbance values were expressed as percentages relative to controls, and the concentrations resulting in 50% inhibition of cell growth (IC_50 _values) were calculated.

### Statistical analysis

Results were presented as means of three independent experiments (± SD). Statistical analyses were performed using SPSS 13.0. Comparisons of optical density values, percentage of viable cells and number of apoptotic cells among groups were performed using the two-tailed Student's *t *test or ANOVA. *P *< 0.05 was considered statistically significant.

## Results

### Knock-down of *AEG-1 *by specific siRNAs

**Figure 1:**
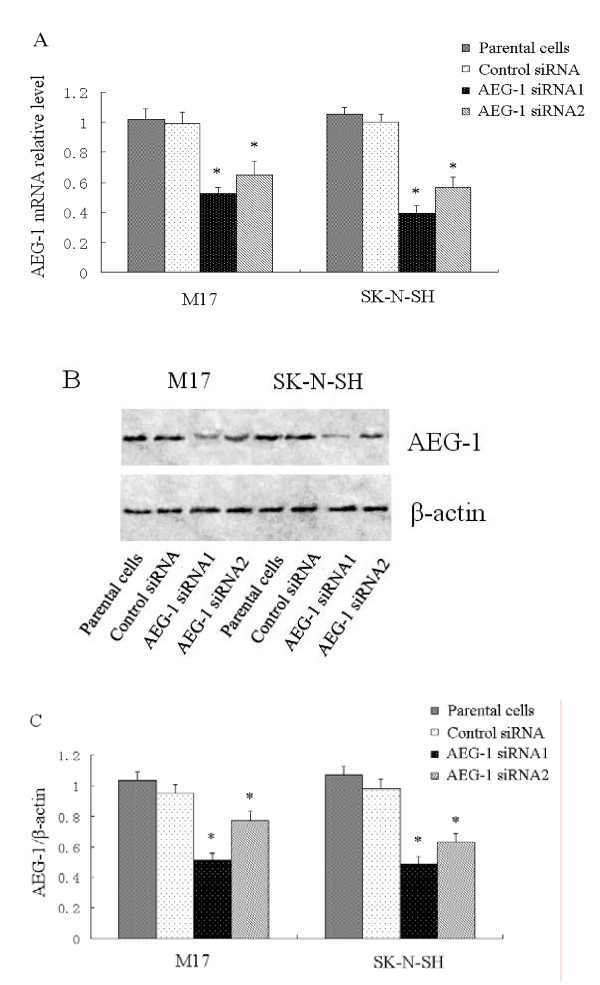
**Knock-down of *AEG-1 *by specific siRNAs**. Fourty-eight hours after transfection, cells were harvested. (A), *AEG-1 *mRNA levels were quantified by real-time PCR analysis. Data were normalized by using GAPDH as an internal standard. *P < 0.05 vs. parental cells. (B, C) *AEG-1 *protein level was analyzed by western blot. β-actin expression was monitored as the internal standard. *P < 0.05 vs. parental cells. These experiments were performed in triplicate.

In order to knock down *AEG-1*, we used two different 21-base pair siRNA constructs: *AEG-1*-siRNA1 and *AEG-1*-siRNA2. As shown in Figure 1, transfected M17 and SK-N-SH with either *AEG-1*-siRNA1 or *AEG-1*-siRNA2 resulted in knock down of *AEG-1 *at both the transcription and translation levels in each neuroblastoma cell lines. Control siRNA transfected cells had no significant impact on *AEG-1 *expression levels compared with parental cells. *AEG-1*-siRNA1 was used to process the follow investigation.

### *AEG-1 *knockdown inhibits proliferation and promotes apoptosis in neuroblastoma cells

**Figure 2:**
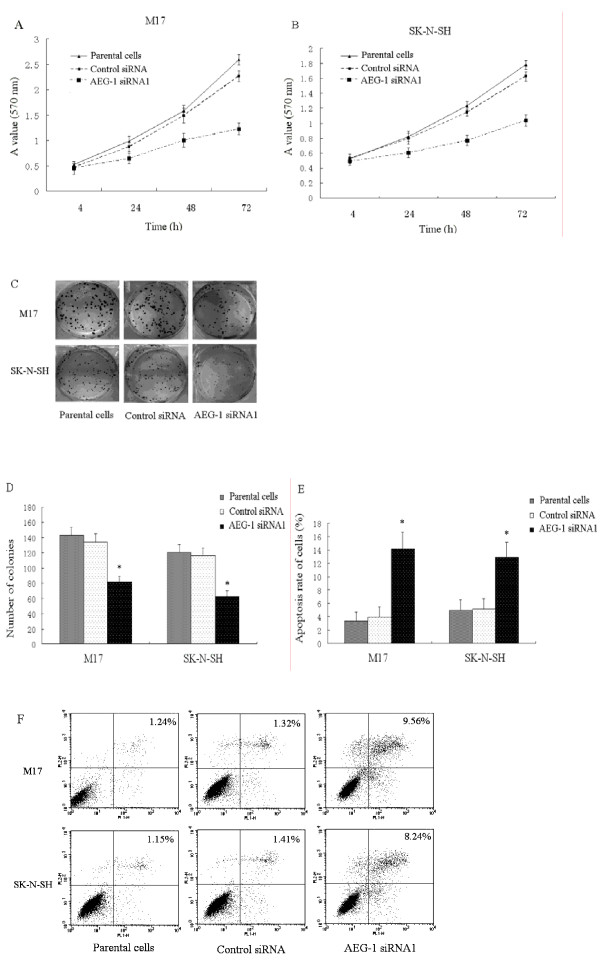
***AEG-1 *knockdown inhibits proliferation and promotes apoptosis in neuroblastoma cells**. (A, B) Cell viability was evaluated by MTT assay. The results of cell proliferation assay showed a significant decrease in the number of cells by 42.9% in M17 and 49.5% in SK-N-SH in 72 h. (C, D) Compared with that seen in the parental cells, the number of colonies was significantly reduced in the *AEG-1 *siRNA1 transfected group (*P < 0.05 vs. parental cells). Each experiment was performed three times in triplicate. (E) The apoptosis rate of *AEG-1 *siRNA1 transfected cells significantly increased by 9.6% ± 1.7% in M17 and 9.0% ± 1.4% in SK-N-SH cells (*P < 0.05 vs. parental cells), respectively. (F) Representative results are shown. These experiments were performed in triplicate.

In order to examine the role of *AEG-1 *on neuroblastoma cell proliferation, we examined the effect of *AEG-1 *siRNA on neuroblastoma cell growth and colonogenic assay. As shown in Figure 2A and 2B, *AEG-1*-siRNA1 significantly decreases cell proliferation by 42.9% in M17 and 49.5% in SK-N-SH at 72 hours compared to control group, respectively. Furthermore, colony forming ability was also affected by transfection with *AEG-1 *siRNA1 (Figure 2C and 2D).

We also evaluated apoptotic levels of neuroblastoma cell lines. As shown in Figure 2E and 2F, the apoptotic cell fraction was 3.75% and 2.9% in control siRNA-transfected M17 and SK-N-SH cells, respectively. In contrast, they were 13.2% and 11.8% in *AEG-1 *siRNA1-transfected cells.

### Knock down of *AEG-1 *accumulates G_0_/G_1_-phase cells

**Figure 3:**
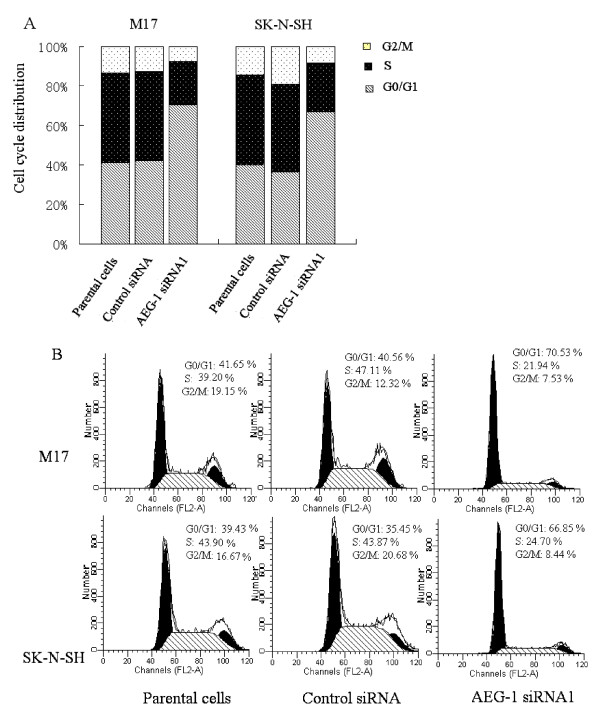
**Knock down of *AEG-1 *reduces S and G2/M-phase cells**. (A) In both M17 and SK-N-SH cells 48 hours after *AEG-1 *siRNA1 transfection, the population of G_0_/G_1 _phase was significantly increased and the population of S phase and G_2_/M phase was obviously decreased (*P < 0.05 vs. parental cells). (B) Representative results are shown. These experiments were performed in triplicate.

Cell proliferation inhibited by knockdown of *AEG-1 *was also shown in other types of mammalian cells [9,10]. To reveal mechanism involved in proliferation inhibition, we analyzed cell cycle by using flow cytometry. As shown in Figure 3, knockdown of *AEG-1 *resulted in accumulation in the G_0_/G_1 _phase and reduction of S and G_2_/M phase cell.

### Knock down of *AEG-1 *sensitize cells to cisplatin and doxorubicin

**Figure 4:**
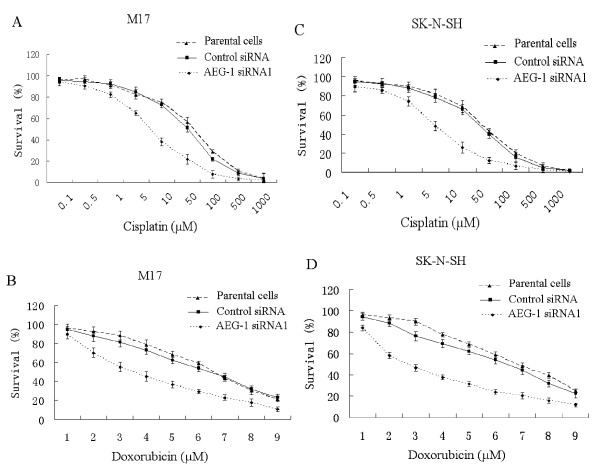
**Knock down of *AEG-1 *sensitized cells to cisplatin and doxorubicin**. M17 and SK-N-SH cells were transfected with *AEG-1 *siRNA1 or control siRNA for 48 h, then exposed to various concentrations of cisplatin or doxorubicin for 48 h, and the viability was accessed. The percentage of cell growth was calculated by comparison of the A570 reading from treated cells versus control cells. The IC_50 _value of M17 cells to cisplatin (A) and doxorubicin (B) was 6.4 and 3.4 μM, respectively. The IC_50 _values of SK-N-SH cells to cisplatin (C) and doxorubicin (D) were 3.3 and 2.8 μM, respectively. These results demonstrated that transfection of *AEG-1 *siRNA1 increased the sensitivity of M17 and SK-N-SH to cisplatin by 4.3- and 4.5-fold and to doxorubicin by 1.9- and 2.3-fold, respectively. Each experiment was performed three times in triplicate.

Except to operation, chemotherapy is also important in treatment of neuroblastoma, especially in neoadjuvant chemotherapy. Here we tested if knock down *AEG-1 *could sensitize neuroblastoma cells to chemotherapeutic agents. M17 and SK-N-SH were exposed to cisplatin and doxorubicin after transfected with *AEG-1*-siRNA1 for 48 hours. Cells' viability was evaluated using a MTT assay. As shown in Figure 4, cells transfected with *AEG*-*1*-siRNA1 were more sensitive to cisplatin and doxorubicin than control. The sensitivities of M17 and SK-N-SH to cisplatin were enhanced by knock down of *AEG-1 *by 4.3- and 4.5-fold and to doxorubicin by 1.9- and 2.1-fold, respectively.

## Discussion

Neuroblastoma is one of the most frequently occurring solid tumors in children, especially in the first year of life, when it accounts for 50% of all tumors. It is the second most common cause of death in children, only preceded by accidents [5]. Despite many advances in the past three decades, neuroblastoma has remained an enigmatic challenge to clinical and basic scientists. Elucidation of the exact molecular pathways of neuroblastoma will enable researchers and clinicians to stratify the disease and adapt therapy to the risk of relapse or progress. A large body of basic research into genes and oncogenes has accumulated up till present.

Increased/decreased expression of the molecular factors, MYCN, H-ras, and trkA is well known in neuroblastoma [1-4]. However, the poor prognosis for advanced neuroblastoma still reflects in part the lack of knowledge about the tumor's basic biology.

Aberrant *AEG-1 *expression has been observed in some solid tumors including breast, brain and prostate [13,14]. Our earlier data have demonstrated that *AEG-1 *expression was increased in human neuroblastoma tissues and cultured cells compared to normal brain tissues. The expression level of *AEG-1 *was correlated with the clinical staging of neuroblastoma. Multivariate analysis suggested that *AEG-1 *might be an independent biomarker for the prediction of prognosis of neuroblastoma (submitted). In our current study, we evaluated the possibility of *AEG-1 *as a therapeutic target of neuroblastoma.

*AEG-1 *has been reported to be upregulated in several malignancies and play a critical role in Ha-*ras*-mediated oncogenesis through the phosphatidylinositol 3-kinase/AKT signaling pathway [15]. Emdad et al. documented that *AEG-1 *is a significant positive regulator of NF-κB [11]. Activation of NF-κB by *AEG-1 *could represent a key molecular mechanism by which *AEG-1 *promotes anchorage-independent growth and invasion, two central features of the neoplastic phenotype. Furthermore, Kikuno et al. revealed that aberrant *AEG-1 *expression as a positive auto-feedback activator of AKT and as a suppressor of FOXO3a in prostatic cancer cells [10]. In this study, we adopted a strategy of RNA interference to inhibit expression of *AEG-1 *in two neuroblastoma cell lines, M17 and SK-N-SH. The results revealed that after transfection with *AEG-1 *siRNA, mRNA level and protein level of the *AEG-1 *gene decreased, and meanwhile cell growth inhibited and apoptosis increased. Therefore, our data also confirmed that *AEG-1 *serves in regulating both cell proliferation and survival. *AEG-1 *knockdown may not only effect the NF-κB signaling pathway, but also the PI3K/AKT signaling pathway, either directly or indirectly and also influences the function of several PI3K/AKT downstream substrates. Our reports also shown that knockdown of *AEG-1 *resulted in accumulation in the G_0_/G_1 _phase and reduction of S and G_2_/M phase cell. The eukaryotic cell cycle is regulated via the sequential activation and inactivation of CDKs that drive cell cycle progression through the phosphorylation and dephosphorylation of regulatory proteins. The underlying mechanisms are still unclear.

Since *AEG-1 *might play important role in neuroblastoma cell growth, we explored the therapeutic role of *AEG-1 *in combination with chemotherapeutic drug. We found that knockdown of *AEG-1 *synergistically enhanced the cytotoxicity of cisplatin and doxorubicin. Cisplatin forms inter- and intra-strand DNA cross-links. The cytotoxic effect was likely a result of inhibition of replication by cisplatin-DNA adducts and induction of apoptosis. Cisplatin is a widely used anticancer agent and frequently applied via transarterial chemo-embolization or systemically in neuroblastoma. Our results suggest that cisplatin chemotherapy could be more effective in combination with RNAi mediated knockdown of *AEG-1*. Clearly, for the development of such a therapeutic strategy for clinical use, a suitable vector system is necessary. These will be further explored in future work.

In summary, our present study suggests that overexpressed *AEG-1 *enhance the tumorogenic properties of neuroblastoma cells. Knockdown of *AEG-1 *could inhibit proliferation and enhance chemo-sensitivity to cisplatin or doxorubicin in neuroblastoma cells and therefore it could be a new adjuvant therapy for neuroblastoma.

## Competing interests

The authors declare that they have no competing interests.

## Authors' contributions

HL and LW carried out cell transfection, immunoblotting analysis; CL and LX contributed to cell transfection, cell treatments, RT-PCR and flow cytometry analysis. HL, XS and RS supervised experimental work and wrote the manuscript. All authors read and approved the final manuscript.

## References

[B1] Castleberry RP (1999). Predicting outcome in neuroblastoma. N Engl J Med.

[B2] Castel V, Garcia-Miguel P, Canete A, Melero C, Navajas A, Ruiz-Jimenez JI, Navarro S, Badal MD (1999). Prospective evaluation of the International Neuroblastoma Staging System (INSS) and the International Neuroblastoma Response Criteria (INRC) in a multicentre setting. Eur J Cancer.

[B3] Castleberry RP, Pritchard J, Ambros P, Berthold F, Brodeur GM, Castel V, Cohn SL, De Bernardi B, Dicks-Mireaux C, Frappaz D, Haase GM, Haber M, Jones DR, Joshi VV, Kaneko M, Kemshead JT, Kogner P, Lee REJ, Matthay KK, Michon JM, Monclair R, Roald BR, Seeger RC, Shaw PJ, Shimada H, Shuster JJ (1997). The International Neuroblastoma Risk Groups (INRG): a preliminary report. Eur J Cancer.

[B4] Shimada H, Ambros IM, Dehner LP, Hata J, Joshi VV, Roald B, Stram DO, Gerbing RB, Lukens JN, Matthay KK, Castleberry RP (1999). The International Neuroblastoma Pathology Classification (the Shimada system). Cancer.

[B5] Chan HS, Gallie BL, DeBoer G, Haddad G, Ikegaki N, Dimitroulakos J, Yeger H, Ling V (1997). MYCN protein expression as a predictor of neuroblastoma prognosis. Clin Cancer Res.

[B6] Gordon SJ, Pearson AD, Reid MM, Craft AW (1992). Toxicity of single-day high-dose vincristine, melphalan, etoposide and carboplatin consolidation with autologous bone marrow rescue in advanced neuroblastoma. Eur J Cancer.

[B7] Su ZZ, Kang DC, Chen Y, Pekarskaya O, Chao W, Volsky DJ, Fisher PB (2002). Identification and cloning of human astrocyte genes displaying elevated expression after infection with HIV-1 or exposure to HIV-1 envelope glycoprotein by rapid subtraction hybridization, RaSH. Oncogene.

[B8] Kang DC, Su ZZ, Sarkar D, Emdad L, Volsky DJ, Fisher PB (2005). Cloning and characterization of HIV-1-inducible astrocyte elevated gene-1, AEG-1. Gene.

[B9] Lee SG, Su ZZ, Emdad L, Sarkar D, Fisher PB (2006). Astrocyte elevated gene-1 (AEG-1) is a target gene of oncogenic Ha-ras requiring phosphatidylinositol 3-kinase and c-Myc. Proc Natl Acad Sci USA.

[B10] Kikuno N, Shiina H, Urakami S, Kawamoto K, Hirata H, Tanaka Y, Place RF, Pookot D, Majid S, Igawa M, Dahiya R (2007). Knockdown of astrocyte-elevated gene-1 inhibits prostate cancer progression through upregulation of FOXO3a activity. Oncogene.

[B11] Emdad L, Sarkar D, Su ZZ, Randolph A, Boukerche H, Valerie K, Fisher PB (2006). Activation of the nuclear factor kappaB pathway by astrocyte elevated gene-1: implications for tumor progression and metastasis. Cancer Res.

[B12] Song X, Liu X, Chi W, Liu Y, Wei L, Wang X, Yu J (2006). Hypoxia-induced resistance to cisplatin and doxorubicin in non-small cell lung cancer is inhibited by silencing of HIF-1alpha gene. Cancer Chemother Pharmacol.

[B13] Brown DM, Ruoslahti E (2004). Metadherin, a cell surface protein in breast tumors that mediates lung metastasis. Cancer Cell.

[B14] Li J, Zhang N, Song LB, Liao WT, Jiang LL, Gong LY, Wu J, Yuan J, Zhang HZ, Zeng MS, Li M (2008). Astrocyte elevated gene-1 is a novel prognostic marker for breast cancer progression and overall patient survival. Clin Cancer Res.

[B15] Lee SG, Su ZZ, Emdad L, Sarkar D, Franke TF, Fisher PB (2008). Astrocyte elevated gene-1 activates cell survival pathways through PI3K-Akt signaling. Oncogene.

